# Lateral Spread Response in Hemifacial Spasm: Physiological Mechanisms, Intraoperative Utility, and Prognostic Significance

**DOI:** 10.7759/cureus.82794

**Published:** 2025-04-22

**Authors:** Anshika Baranwal, Mahesh Arjundan Gadhvi, Mohit Agrawal, Shival Srivastav, Abhinav Dixit

**Affiliations:** 1 Physiology, All India Institute of Medical Sciences, Jodhpur, Jodhpur, IND; 2 Neurosurgery, All India Institute of Medical Sciences, Jodhpur, Jodhpur, IND

**Keywords:** abnormal muscle response, hemifacial spasms, intraoperative neuromonitoring (ionm), lateral spread response, microvascular decompression (mvd)

## Abstract

Facial nerve compression by blood vessels near the brainstem can cause hemifacial spasm (HFS). There are two treatment options for this condition: botulinum toxin and surgical microvascular decompression (MVD). During microvascular decompression, the facial nerve is separated from the offending vessel, and intraoperative neuromonitoring in these patients demonstrates abnormal muscle response (AMR), which is known as the lateral spread response (LSR). Though the disappearance of lateral spread response is a hallmark of successful microvascular decompression, little information is available about its physiological origin and diagnostic utility. In the present review, we have attempted to address the aforementioned caveats about lateral spread response with an emphasis on the intraoperative utility and diagnostic role of this electrophysiological phenomenon.

## Introduction and background

Hemifacial spasm (HFS) presents as involuntary facial twitching on the unilateral side of the face, primarily beginning from the orbicularis oculi muscle. It is caused by the compression of the facial nerve at its exit point from the brainstem by blood vessels.

Earlier in the mild cases, the treatment involved medical management in the form of oral medications such as carbamazepine, clonazepam, baclofen, and gabapentin, but nowadays, botulinum toxin injections are used for standard care [1]. Patients who are refractory to medicine or botulinum toxin treatment are candidates for surgical treatment, wherein they undergo a procedure known as microvascular decompression (MVD), which entails the separation of the vascular compression between the artery and nerve from the intracranial route [2]. Identifying the correct offending vessel in a restricted space of the cerebellopontine angle, where there may be more than one vessel impinging on/lying near the facial nerve, can sometimes become challenging. Here, the neuromonitoring phenomenon known as the lateral spread response (LSR) can be helpful.

Lateral spread response (LSR)

The zygomatic branch of the facial nerve innervates the orbicularis oculi muscle, and the marginal mandibular branch controls the mentalis muscle. Under normal physiological conditions, the stimulation of one branch of facial nerve produces electromyography (EMG) changes in only the muscles supplied by the same branch; in this case, zygomatic branch stimulation must produce response in only orbicularis oculi muscle; conversely, in patients with HFS, the stimulation of a single facial nerve branch results in the generation of responses in both the orbicularis oculi and mentalis muscles, suggestive of the LSR.

**Figure 1:**
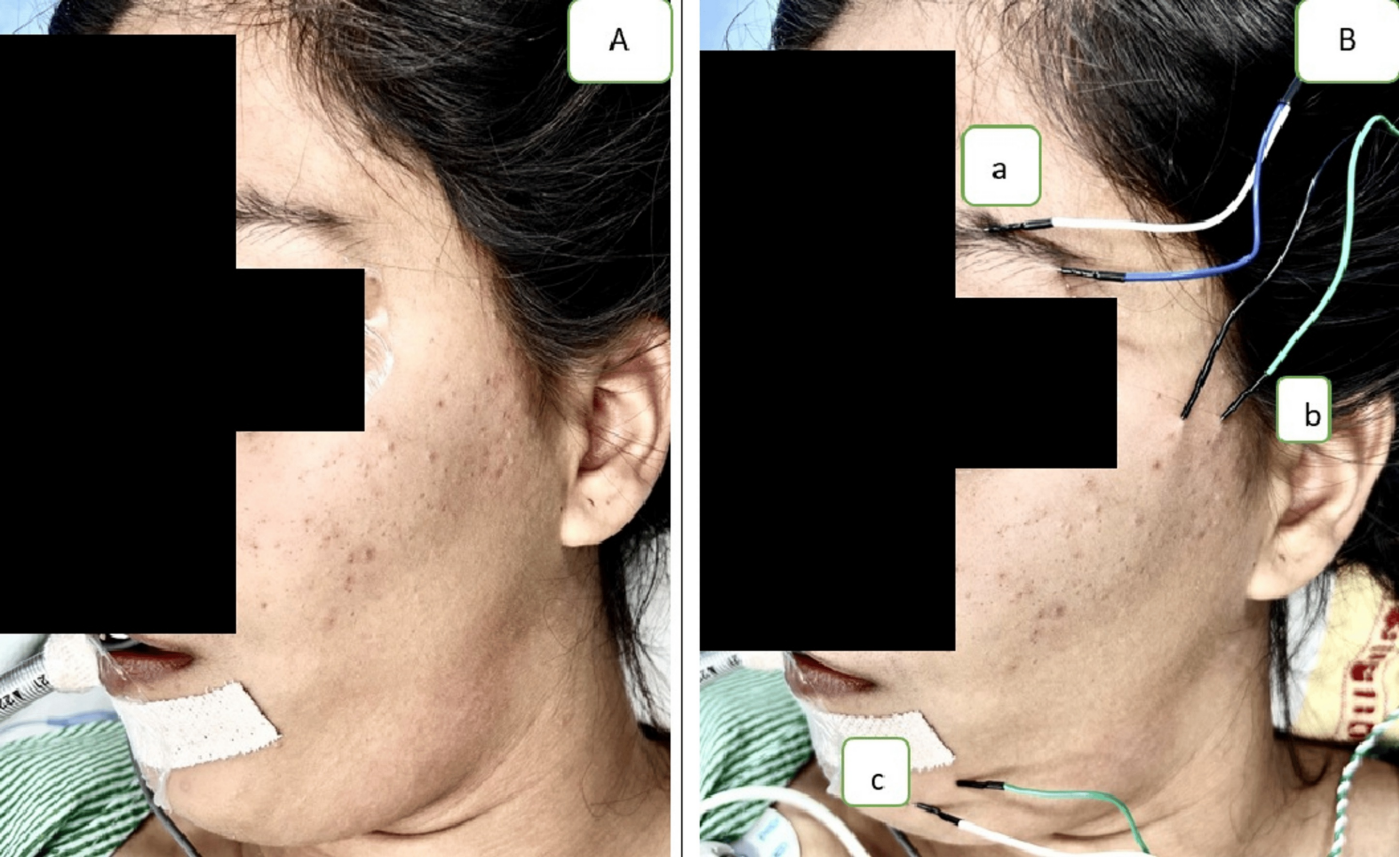
Reference image showing the placement of electrodes on the patient (A) The face of the patient before electrode placement. (B) Recording electrodes are placed on the orbicularis oculi (a) and mentalis muscles (c) and a pair of stimulating electrodes over the zygomatic branch of the facial nerve (b)

**Figure 2:**
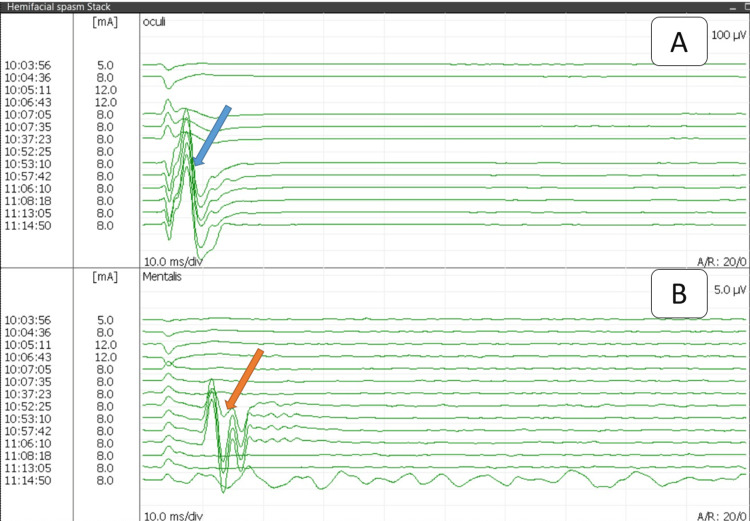
Reference image of stack view showing responses from the orbicularis oculi (A) and mentalis muscles (B) after stimulating the zygomatic branch of the facial nerve Response started once the muscle relaxant was weaned off in both muscles, showing normal response in the orbicularis oculi (blue arrow) and abnormal muscle response in the mentalis muscle (orange arrow). After MVD at 11:08 AM, the normal response in the oculi remained the same, while the abnormal muscle response from the mentalis muscle disappeared. Recording by Medtronic NIM-ECLIPSE E4 SD™ system (Minneapolis, MN) MVD: microvascular decompression

The detection and monitoring of LSR during surgical interventions are crucial for assessing the efficacy of procedures to relieve HFS symptoms. Successful decompression is monitored by the attenuation or eradication of LSR, signifying normalized nerve function after vascular decompression (Figure 1 and Figure 2).

## Review

Abnormal muscle response (AMR) during a surgical procedure was first documented by Møller and Jannetta [3]. In their pioneering work, they stimulated the marginal mandibular and zygomatic branches of the facial nerve. They took recordings from the mentalis and orbicularis oculi muscles of the patient with HFS. They observed that regardless of the specific branch of the facial nerve stimulated, the response was generated in both muscles, which disappeared after the decompression procedure. They proposed that this could be due to antidromic stimulation-induced cross-transmission.

In a subsequent study conducted by Møller and Jannetta, AMRs were recorded from the mentalis muscle in the cohort of 67 patients with HFS [4]. Just after induction, they recorded an early component of the response at 10 ms intervals, followed by afterdischarges that disappeared after dura opening. Notably, only a single AMR at 10 ms persisted at the mentalis. Interestingly, variations were observed among patients, where in some cases, AMR disappeared immediately after dura opening, while in others, it vanished following decompression. Of significance, in 24 out of 67 patients, AMR persisted postoperatively. Among these, four patients displayed postoperative AMR amplitudes identical to the preoperative levels, and these particular cases did not show symptomatic improvement. In all 43 patients in whom AMR disappeared during operation, there were no postoperative symptoms. Sen and Møller extended their investigations to animal models, demonstrating positive LSR in rats with damaged facial nerve by stimulation near the brainstem [5].

After these pioneering studies, subsequent studies were done to find the intraoperative role of LSR during MVD in HFS. Isu et al. conducted a comprehensive study and found that out of 40 patients operated on for MVD in HFS, all the patients had LSR present intraoperatively, which disappeared after decompression in 38 patients [6]. In the remaining two patients, LSR persisted postoperatively. Interestingly, these patients experienced a continuation of symptoms despite the surgical intervention. This finding highlights the potential utility of LSR as an intraoperative marker indicative of the efficacy of MVD. The same notion is supported by many other studies [7-9].

In one of the most extensive series to date, Kong et al. sought to evaluate the prognostic significance of the disappearance of LSR over a one-year follow-up period in a cohort of 263 patients diagnosed with HFS [10]. Among the cohort, LSR was initially recorded in all 263 patients. During the surgical procedure, LSR disappeared in 230 patients, and notably, 208 patients experienced complete relief from pain, while 22 patients reported partial relief within the first year of follow-up. In 33 patients in whom LSR persisted during decompression, 22 patients had a complete relief of symptoms within one year of follow-up, while in 11 patients, symptoms persisted.

These findings show the effective role of LSR during the MVD of HFS patients. The same results are also supported by recent literature [11,12].

Is the time of the disappearance of LSR important?

The timing of the disappearance of LSR during MVD surgery for HFS was scrutinized by Kim et al. [13]. This research involved the comparison of LSR disappearance occurrences before and after MVD in a cohort of 273 patients. In 90 cases, LSR disappeared before MVD, and in these patients, 75.8% were symptom-free at the one-year follow-up. While LSR disappeared after decompression in 183 patients, 92.9% showed complete relief from symptoms in the one-year follow-up period. These findings emphasize the significance of the timing of the disappearance of LSR in affecting the outcome. A study also showed that the disappearance of LSR before MVD was associated with smaller compressing vessels, spacious posterior fossa, and mild nerve deviation [14].

A similar study was carried out by Kang et al., where in 128 out of 199 patients, LSR disappeared after MVD, whereas in 71, LSR disappeared before MVD [15]. In the former cohort, relief from pain was achieved in 78.9% and 83.6% of patients within the one-year and two-year follow-up periods, respectively, while in the latter cohort in which LSD disappeared before MVD, pain relief was achieved in only 47.9% and 52.1% of patients, at the one-year and two-year follow-up, respectively. This study reinforces that the disappearance of LSR after decompression exhibits superior predictability compared to cases where LSR vanishes before MVD.

Some novel studies investigating the predictability of LSR

In a recent investigation by Cho et al., the significance of intraoperative lateral spread response (IO-LSR) disappearance and postoperative day 2 (POD2) LSR disappearance was assessed in relation to treatment outcomes [16]. They found a 98% cure rate after the disappearance of LSR in IO-LSR and POD2 LSR. This observation implies that measuring LSR at both stages of the surgical process enhances the predictive accuracy of treatment outcomes. Kim et al. examined the prognostic value of LSR one month after surgery [17]. They discovered that the presence of LSR had a poor predictive value after one year of follow-up but could be helpful in the early detection of patients who would not do well. These studies highlight the importance of assessing LSR during surgery, the early postoperative period, and the following months.

Additionally, Miao et al. introduced the concept of multibranch AMR during HFS, where adding more muscles in recording increased the chances of getting AMR during surgery [18]. Only the mentalis produces AMR in 74% of patients, while incorporating the frontalis and orbicularis oris muscles improved the rate of AMR generation up to 86.5%. Further, adding the platysma increased the rate to 98.4%.

Some studies have recently been carried out to see whether the pattern of LSR waveform can also affect the postoperative outcomes. They categorized the recording waveform under monophasic or polyphasic AMRs based on the number of peaks, where more than three peaks were defined as a polyphasic response. They observed that the initial polyphasic response in LSR had the persistence of symptoms in 56.5% of patients at one week after surgery and 43.5% of patients at one month after surgery. They also found that the anterior cerebellar artery was the cause of most polyphasic responses [19]. In a subsequent study by the same authors, the stimulation of the temporal and marginal mandibular branches was also compared. They found the stimulation of the temporal branch with recording AMR from the mentalis more useful than the stimulation of the marginal mandibular branch and recording AMR from the orbicularis oculi [20].

Questionable role of LSR disappearance in prediction

Though there are numerous studies demonstrating the predictability of symptom-free outcomes after the disappearance of LSR in HFS patients, some studies have shown contradictory results. A limited role for LSR disappearance in HFS operations has been reported by several researchers. In a study by Kiya et al., out of 38 patients with HFS, LSR vanished in 21 patients [21]. Among those with LSR disappearance, two patients experienced moderate postoperative symptoms that persisted. However, six out of the 17 patients with intact LSR experienced postoperative symptoms. All 38 patients, regardless of the status of LSR, were symptom-free after three months. These findings suggest that the elimination of LSR may not show a clear-cut association with postoperative prognosis. Similar outcomes were obtained by Hatem et al., where all patients were symptom-free after three months postoperatively, irrespective of the LSR status [22].

The study conducted by Joo et al. adds further complexity to the understanding of the prognostic value of LSR disappearance in the context of MVD for HFS [23]. They stimulated the zygomatic branch and obtained recordings from the mentalis for LSR; however, after a six-month follow-up period, they discovered conflicting findings that suggested the disappearance of LSR did not have a good prognostic value in cases with pain relief in patients with persistent LSR after MVD.

Thirumala et al. observed that LSR disappearance had good prognostic value just after follow-up or within three months after surgery [24]. However, their findings indicated that after one year, the status of LSR did not impact the prognostic outcomes, suggesting that the predictive power of LSR might be more prominent in the early postoperative period. On the other hand, El Damaty et al. mentioned that LSRs may only help an adequate decompression during surgery but failed to represent treatment outcome [25]. We found similar results in other studies [26-28]. These imply that there is a long way to go before the involvement of LSR in HFS patients is confirmed.

Mechanism of LSR generation: A major controversy

The exploration of the mechanism underlying AMR in the context of HFS has been a subject of scientific inquiry since its initial recording. According to the central cause theory and peripheral cause theory, which were proposed in 1996 and 1997, respectively, LSR may be caused by either the hyperactivity of the facial nuclei or aberrant connections between various facial nerve fibers.

Central Cause Theory

To identify the reason behind LSR, Ishikawa et al. studied the F-wave in patients with HFS [29]. F-waves are generated when electrical stimulation is applied to a nerve, travels back to the nuclei, and sends signals back to the muscles. The researchers observed notable differences in F-wave characteristics between the affected and healthy sides in patients with HFS. They found that the F-wave had a larger amplitude and shorter latency than the healthy side, suggesting a central cause due to the higher susceptibility of the facial motor nucleus.

The study conducted by Wilkinson and Kaufmann sought to investigate the potential causes of LSR by examining facial nerve motor evoked potentials (MEPs) [30]. Facial motor evoked potential is generated by stimulating the motor cortex through transcranial stimulation and recording from facial muscles. They found that during MVD, LSR disappeared along with decreased facial muscle MEP latency and amplitude, suggesting the central cause of injury.

Møller et al. focused on a similar response to LSR in patients with hemilingual spasms, where the hypoglossal nerve is compressed by the artery [31]. In these patients, MEP during MVD revealed a late response in tongue muscles at around 40 ms, which suggested an abnormal lingual nerve motor nucleus. The response disappeared after MVD, suggesting the same theory for HFS. The same kind of case was also reported by Barkyoumb et al., where hemilingual spasm was due to a compressed nerve by the posterior inferior cerebellar artery. MVD in the patient produced recovery from the symptoms [32].

Wilkinson et al. investigated the effect of different anesthetic agents in LSR [33]. The researchers compared the effects of only total intravenous anesthesia (TIVA) with TIVA in combination with desflurane, an inhalational anesthetic drug. Desflurane specifically affects the corticobulbar tract while sparing peripheral nerve transmission. The study revealed that when desflurane was introduced in conjunction with TIVA, there was a significant reduction in the amplitude of LSR compared to TIVA alone, indicating that a central cause is primarily responsible for LSR in HFS patients. Moreover, the same authors extended their investigation to MEPs in HFS under the influence of desflurane and found the same results, suggesting the central cause of the disease [34].

A similar study was carried out by Yang et al., showing that the effect of adding sevoflurane with TIVA can affect AMR amplitude. This also supports the central cause theory [35].

Peripheral Cause Theory

The other theory contends that hyperexcitable facial nerve motor neurons should not cause LSR. The disappearance of LSR following facial nerve decompression indicates that the cause should be at the site of injury. In order to pinpoint the source, Yamashita et al. employed a double stimulation approach during LSR production to investigate the source and underlying mechanism [36]. According to them, the LSR remained constant during double train stimulation, and the response amplitude was the same for both stimulations, showing that LSR is not generated due to motor neuronal hyperexcitability.

The study's results add complexity to the ongoing debate regarding the central versus peripheral origins of LSR. While the central cause theory has been suggested by many researchers, the controversy persists, with studies such as Yamashita et al.'s providing evidence that challenges a straightforward interpretation of LSR as solely a result of motor neuron hyperexcitability.

Some of the recent studies proposed that the demyelination of facial nerves due to constant irritation can be the reason behind AMR [19,20]. Polyphasic response is the same as the EMG response seen in patients with demyelinating neuropathy.

Can only the artery be the cause of LSR?

HFS could also be due to vascular abnormalities other than an artery, such as compression due to an aneurysm or a vein affecting the nerve, and the role of LSR in such cases was also studied. Lee et al. systematically examined LSR, where the causal factor for HFS was the aneurysm of the artery, and MVD caused symptomatic relief, as well as the disappearance of LSR [37]. A similar case of HFS due to a vertebral artery aneurysm has also been reported [38]. Eun et al. presented a noteworthy case study elucidating HFS due to a venous compression rather than an arterial involvement [39]. A case was reported by Al-Mutawa and Schroeder regarding HFS due to an arachnoid band. They found that the primary cause of HFS was an arachnoid band without any vessel involvement. LSR disappeared after cutting the band, and the patient was symptom-free in follow-up [40].

Novel methods combined with LSR for HFS

The use of new intraoperative neurophysiological monitoring (IONM) modality such as Z-L response (ZLR) combined with LSR is being investigated. In ZLR, the offending vessel is stimulated near the site of compression, and the spread of current from the vessel to the nerve is recorded in an F-wave-like pattern. Combining ZLR with LSR can improve monitoring and help reduce morbidity and mortality [41]. ZLR alone cannot be more beneficial than LSR, but it can help to find the actual offending vessel [42].

A distinct EMG recording from facial muscles was observed by Cho et al. during the placement of Teflon between the root exit zone and the facial nerve, named "Sang-ku sign" (SKS). They found a significant correlation between the presence of SKS and the disappearance of LSR and proposed the utilization of SKS with LSR for future monitoring [43].

Diagnostic value of LSR

The study conducted by Park et al. evaluated the diagnostic value of LSR for HFS and found that though 13 patients with symptoms of HFS had absent LSR in the preoperative phase, the presence of vessels near the nerve in MRI and the relief in pain after surgery suggested no diagnostic role for it [44].

Anesthesia

From its use for the first time, neuromuscular blockers are only given at induction during LSR recording, and throughout surgery, TIVA is recommended. However, there has been successful exploration of the use of partial neuromuscular blockers throughout LSR recording [45]. To find the effect of inhalational anesthesia with TIVA during LSR recording, Yang et al. added sevoflurane with propofol+remifentanil and found that sevoflurane with a <0.5 minimal alveolar concentration with TIVA does not affect AMR amplitude, which can be used during LSR recording [35].

## Conclusions

LSR recording may be a valuable neuromonitoring technique during MVD for HFS. However, there are many unanswered questions related to its generation, as well as its role in predicting outcomes. Novel methods and approaches are being explored to enhance our understanding of LSR and its potential role. We hope that further work in this domain will help us understand the origins and further establish the role of LSR as a neurophysiological marker.
